# Ertapenem-Induced Encephalopathy in a Patient With Normal Renal Function

**DOI:** 10.1177/2324709616689376

**Published:** 2017-01-01

**Authors:** S. Scott Sutton, Mark Jumper, Sean Cook, Babatunde Edun, Michael D. Wyatt

**Affiliations:** 1 University of South Carolina, Columbia, SC, USA; 2 Dorn Veterans Affairs Medical Center, Columbia, SC, USA

**Keywords:** drug-induced encephalopathy, ertapenem, drug-induced neurotoxicity

## Abstract

Drug-induced neurotoxicity is a rare adverse reaction associated with ertapenem. Encephalopathy is a type of neurotoxicity that is defined as a diffuse disease of the brain that alters brain function or structure. We report a patient with *normal* renal function who developed ertapenem-induced encephalopathy manifesting as altered mental status, hallucinations, and dystonic symptoms. The patient’s symptoms improved dramatically following ertapenem discontinuation, consistent with case reports describing ertapenem neurotoxicity in renal dysfunction. Since clinical evidence strongly suggested ertapenem causality, we utilized the Naranjo Scale to estimate the probability of an adverse drug reaction to ertapenem. Our patient received a Naranjo Scale score of 7, suggesting a probable adverse drug reaction, with a reasonable temporal sequence to support our conclusion.

## Introduction

Drug-induced neurotoxicity is a rare adverse reaction associated with carbapenem antibiotics.^1-3^ The data demonstrating a neurotoxic association for carbapenems stems from animal models, observational analyses, randomized controlled trials, meta-analysis, and case reports.^1-3,7-14^ Imipenem is considered to have the highest incidence of neurotoxicity among carbapenems, although there are published literature that refute this consensus.^3,7,15-19^ Encephalopathy is a type of neurotoxicity that is defined as a diffuse disease of the brain that alters brain function or structure. Encephalopathy is characterized by an altered mental state with additional symptoms including progressive loss of memory and cognitive ability, personality changes, myoclonus, nystagmus, tremor, and seizures. Carbapenem neurotoxicity is often evaluated and reported in terms of seizures, and data on encephalopathy are limited. There are case reports of ertapenem neurotoxic effects consisting of encephalopathy in patients with end-stage renal disease or mild renal dysfunction. We report a patient with normal renal function who developed ertapenem-induced encephalopathy manifesting as altered mental status, hallucinations, and dystonic symptoms.

## Case Description

A 67-year-old male with a history of normal cognitive function was admitted with complaints of hallucinating for approximately 1 week. Past medical history was significant for chronic lumbar spine methicillin-sensitive *Staphylococcus aureus* osteomyelitis maintained on life-long cephalexin suppression (discontinued 2 weeks before presentation), non-Hodgkin’s lymphoma in remission for 20 years, hypertension, atrial fibrillation, type 2 diabetes for 13 years (current HgA1C 8.2), ankylosing spondylitis, and esophagitis. Table 1 lists the medication history at hospital admission. The patient’s chronic lumbar spine osteomyelitis led to numerous surgical manipulations of the spinal region, the last of which was in 2012. The spine site became reinfected in 2015 requiring hardware removal and multiple surgical debridements. The spinal abscess wound cultures were positive for *Enterobacter* sp, *Lactobacillus* sp, and *Candida glabrata.* Treatment was initiated with oral fluconazole 600 mg daily, piperacillin/tazobactam 4.5 g every 6 hours (changed to ertapenem 1 g daily), and a wound vacuum-assisted closure dressing placed over the surgical site. Encephalopathic symptoms including hallucinations began 5 days after ertapenem was started. Clinical examination at the bedside revealed the patient to be responsive and alert but clearly lethargic with clouded sensorium, lip smacking, mild asterixis and tremor, and incoherent speech. Through the entire hospitalization the patient remained afebrile with normal vital signs. Leukocyte count remained normal at an average of 5.5 × 10^9^/L (6 readings). His renal function remained stable and electrolytes were normal, as were random glucose checks, thyroid function test, and ammonia level (Table 2). Calculated creatinine clearance by the Cockcroft-Gault equation was 66 mL/min on admission (Table 2). Urine and repeated blood culture sets were negative. Urinalysis was unremarkable. Arterial blood gas was noncontributory. Chest radiograph revealed a left upper lobe pulmonary nodule but subsequent thoracic computed tomography scan with contrast showed no contributing pathology and was stable. Computed tomography of the head revealed no acute intracranial pathology. Electroencephalogram was also normal. An extensive literature review of ertapenem, neurotoxicity, and encephalopathy yielded case reports of ertapenem-induced central nervous system (CNS) events. Ertapenem was stopped and ceftazidime 2 g every 8 hours was started. His hallucinations and related neurological symptoms began to improve and the patient recovered to his baseline mental status within 2 days.

**Table 1.** Medication History at Time of Admission.

Atorvastatin 80 mg daily
Cephalexin 500 mg every 12 hours^a^
Diltiazem 120 mg daily
Ertapenem 1 g daily^b^
Fluconazole 600 mg daily
Gabapentin 300 mg 3 times per day as needed
Glipizide 10 mg twice daily
Metformin 500 mg twice daily
Metoprolol tartrate 25 mg twice daily
Rivaroxaban 20 mg daily

a Cephalexin was discontinued 2 weeks priors to presentation for altered mental status.

b Ertapenem was administered intravenously; all other medications were orally administered.

**Table 2.** Summary of the Blood Test Results of Our Patient on Admission.

Parameters	Readings	Normal Ranges
White cell count	6.8	3.6-11.1 K/mm^3^
Hemoglobin	10.5	12.9-16.1 g/dL
Platelet	476	165-353 K/mm^3^
Sodium	136	135-145 mmol/L
Potassium	4.3	3.5-5.1 mmol/L
Blood urea nitrogen	16	7-26 mg/dL
Creatinine	1.2^a^	0.5-1.3 mg/dL
Calcium	9	8.4-10.2 mg/dL
Albumin	2.2	3.5-5.0 g/dL
Total bilirubin	0.3	0.2-1.2 mg/dL
Alkaline phosphatase	107	40-150 U/L
Alanine aminotransferase	102	0-55 U/L
Aspartate aminotransferase	76	5-34 U/L
Ammonia	29	18-72 Umol/L
Thyroid-stimulating hormone	5.93	0.35-4.94 µIU/mL
Free T4	0.95	0.70-1.48 ng/dL

a The serum creatinine ranged from 1.1 to 1.2 mg/dL over 2 years prior to admission. The creatinine clearance calculation by the Cockcroft-Gault method was 66 mL/min using adjusted body weight (actual body weight 92 kg; adjusted body weight 78 kg; ideal body weight 68 kg). Regardless of the weight utilized (ideal, actual, or adjusted), the patients ertapenem dose of 1 g daily is correct based off creatinine clearance dosing recommendations.^19^

## Discussion

Our report demonstrates a case of ertapenem-induced encephalopathy manifesting as hallucinations and altered mental status. The patient’s symptoms improved dramatically following ertapenem discontinuation, consistent with case reports describing ertapenem neurotoxicity in renal dysfunction. No other medication regimen changes were done during the hospitalization, and no medical history was identified as a cause of the encephalopathy. Since clinical evidence strongly suggested ertapenem causality, we utilized the Naranjo Scale to estimate the probability of an adverse drug reaction to ertapenem.^20^ Our patient received a Naranjo Scale score of 7, suggesting a probable adverse drug reaction, with a reasonable temporal sequence to support our conclusion (Table 3).

**Table 3.** Naranjo Adverse Reaction Probability Scale.

Question	Yes	No	Do Not Know	Case Report Score
Are there previous conclusive reports about the reaction	+1	0	0	+1
Did the adverse event appear after the suspected drug was administered?	+2	−1	0	+2
Did the adverse reaction improve when the drug was discontinued or a specific antagonist was administered?	+1	0	0	+1
Did the adverse event reappear when the drug was re-administered?	+2	−1	0	0
Are there alternative causes (other than the drug) that could on their own have caused the reaction?	−1	+2	0	+2
Did the reaction reappear when a placebo was given?	−1	+1	0	0
Was the drug detected in blood (or other fluids) in concentrations known to be toxic?	+1	0	0	0
Was the reaction more severe when the dose was increased or less severe when the dose was decreased?	+1	0	0	0
Did the patient have a similar reaction to the same or similar drugs in any previous exposure?	+1	0	0	0
Was the adverse event confirmed by any objective evidence?	+1	0	0	+1
Total score = 7^a^				

a Total score 5 to 8 = probable adverse drug reaction, with a reasonable temporal sequence to support the reaction.

Imipenem has demonstrated the highest neurotoxic adverse reaction rates among the carbapenems, while drug-induced encephalopathy is rare with ertapenem and other carbapenems. Imipenem neurotoxicity rates in adults range from 0.4% to 10%, compared to less than 1% for meropenem, doripenem, and ertapenem.^3^ Specifically for ertapenem, postmarketing literature documenting ertapenem neurotoxicity is scarce and limited to case reports with patient-related factors (eg, renal dysfunction, history of central nervous system disorders).^10,11,21-25^ Published case reports include the following:

- Duquaine et al reported 2 patients who developed unusual mental status changes while receiving ertapenem 1 g daily.^21^ One patient had acute renal dysfunction prior to starting therapy. Symptoms resolved on drug discontinuation for both patients.
- Wen et al reported 2 patients with advanced renal failure who received ertapenem at 500 mg daily and subsequently developed acute prolonged neurotoxicity.^22^ Baseline mental function returned 2 weeks after discontinuation of therapy for both patients.
- Lee et al reported 4 patients on hemodialysis that developed unexplained central nervous system toxicity and hallucinations after receiving 500 mg.^23^ Discontinuing the drug led to symptom resolution in all 4 patients.
- Shea et al reported a patient with moderate renal impairment who developed encephalopathy with delayed recovery of 2 weeks. The patient exhibited a full recovery after cessation of ertapenem.^24^
- Oo et al reported 2 cases of ertapenem neurotoxicity, including psychosis and encephalopathy.^10^ Symptoms resolved within 3 and 10 days of discontinuing ertapenem.
- Kong et al reported a case of ertapenem-induced visual hallucinations.^11^ Subsequent re-introduction of ertapenem resulted in almost identical symptoms. Complete resolution of symptoms occurred after discontinuation of ertapenem, as observed in our case study.

Risk factors for carbapenem neurotoxic adverse reactions include the following: (1) basicity strength of the C-2 amino group for carbapenems; (2) accumulation in the CNS, specifically in cases of excessive dosage or impaired renal clearance; (3) patients with CNS disorders (eg, past medical history of seizure disorder).^5,25-27^ Among these risk factors, molecular structure is the most important compound-related factor and renal insufficiency is the most important patient-related factor predisposing to carbapenem neurotoxicity. Pharmacokinetic-related factors may also influence the neurotoxic reactions related to ertapenem. Neurotoxicity risk factors include the following:

- *Compound related*: Carbapenems have discordant rates of neurotoxic adverse reactions potentially because of structural differences within side chains.^1-3,6-9^ Available data demonstrate the probable neurotoxic mechanism is via interactions with the γ-amino butyric acid receptor A (GABA_A_).^8,9^ This interaction is through the side chain on the second carbon atom (C-2) in the carbapenem nucleus. A carbapenem with a basic C-2 side chain increases the binding to GABA_A_ resulting in higher neurotoxic activity in animal models. Because of a basic C-2 side chain, imipenem has a higher tendency to produce neurotoxic adverse reactions compared to meropenem because of the less basic side C-2 side chain of meropenem.^1-3,6-9^ Conversely, ertapenem has an acidic carboxyl group at the C-2 position, is expected to have the lowest binding to GABA_A_, and subsequently is thought to have the lowest potential for neurotoxic activity.
- *Patient related*: Patient-related risk factors for carbapenem neurotoxicity include advanced age, history of CNS disease, renal insufficiency, low body weight, and concurrent use of drugs that are nephrotoxic or that may lower the seizure threshold.^4,5^ Published literature demonstrates that renal dysfunction (end-stage renal disease or mild renal dysfunction) is the primary patient-related factor.
- *Pharmacokinetic related*: There are pharmacokinetic parameters of ertapenem that also could contribute to the drug’s ability to affect the CNS. These factors include a high volume of distribution, high lipophilicity, high protein binding, strong CNS penetration, and metabolites that can also inhibit GABA receptors.^8,9^

Our patient is unique because he had no baseline cerebrovascular disease or history of renal dysfunction. Characteristics of our patient that may have contributed include his age (67), low albumin levels, potential overestimation of his renal function, and prior cephalexin suppression. Ertapenem is highly protein bound—up to 95% bound to albumin; thus, our patient may have been exposed to excessive free drug as his albumin was low. In addition, ertapenem is renally excreted, up to 80%, requiring dosage adjustments for creatinine clearance of less than 30 mL/min. Our patient’s creatinine clearance could have been overestimated as serum creatinine measurements are not as accurate in the elderly.^28^ The creatinine clearance calculation of 66 mL/min utilized the adjusted body weight of the patient. Regardless of the weight utilized (ideal, actual, or adjusted), the patients ertapenem dose of 1 g daily is correct based off creatinine clearance dosing recommendations (Table 2). Furthermore, while there is no known interaction between cephalexin and ertapenem, it is possible that cephalexin suppression could have had an impact on this case. However, the patient tolerated the cephalexin while receiving and tolerated ceftazidime after developing the CNS symptoms. Furthermore, cephalexin was discontinued 2 weeks prior to presentation; therefore, it is unlikely that cephalexin has a role in the symptoms.

Previous studies and reviews on carbapenem antibiotics indicated that the high degree of neurotoxicity is not a class phenomenon for carbapenems, and that there are carbapenems that have a lower degree of neurotoxicity. Therefore, non-imipenem carbapenems are thought to be safely administered at high doses and are useful in the treatment of CNS infections. While this may be true, non-imipenem carbapenems have demonstrated a neurotoxic potential, and our case report demonstrates a carbapenem with an acidic carboxyl group administered to a patient with no neurotoxic risk factors can still develop encephalopathy. Our case report aims to add to the growing body of literature of ertapenem-induced neurotoxicity with the goal of increasing education regarding the potential for these neurotoxic effects.

## Conclusion

We are sharing our experience of a patient with normal renal function who developed neurotoxicity following standard dosing of ertapenem. The goal of this case report is to draw attention to the neurologic and encephalopathic potential of ertapenem. Identifying ertapenem as a potential causative agent of these conditions can spare unnecessary investigations and health care expenditures. In our patient, supportive care following discontinuation of ertapenem resulted in complete resolution of his hallucinations and altered mental status.
